# HIV-1 Vaccine-Induced T-Cell Reponses Cluster in Epitope Hotspots that Differ from Those Induced in Natural Infection with HIV-1

**DOI:** 10.1371/journal.ppat.1003404

**Published:** 2013-06-20

**Authors:** Tomer Hertz, Hasan Ahmed, David P. Friedrich, Danilo R. Casimiro, Steven G. Self, Lawrence Corey, M. Juliana McElrath, Susan Buchbinder, Helen Horton, Nicole Frahm, Michael N. Robertson, Barney S. Graham, Peter Gilbert

**Affiliations:** 1 Statistical Center for HIV Research and Prevention, Vaccine and Infectious Disease Division and the HIV Vaccine Trials Network, Fred Hutchinson Cancer Research Center, Seattle, Washington, United States of America; 2 Merck Research Laboratories, West Point, Pennsylvania, United States of America; 3 HIV Research Section, San Francisco Department of Public Health, San Francisco, California, United States of America; 4 Viral Vaccine Program, Seattle Biomedical Research Institute, Seattle, Washington, United States of America; 5 Vaccine Research Center, National Institute of Allergy and Infectious Diseases, National Institutes of Health, Bethesda, Maryland, United States of America; Emory University, United States of America

## Abstract

Several recent large clinical trials evaluated HIV vaccine candidates that were based on recombinant adenovirus serotype 5 (rAd-5) vectors expressing HIV-derived antigens. These vaccines primarily elicited T-cell responses, which are known to be critical for controlling HIV infection. In the current study, we present a meta-analysis of epitope mapping data from 177 participants in three clinical trials that tested two different HIV vaccines: MRKAd-5 HIV and VRC-HIVAD014-00VP. We characterized the population-level epitope responses in these trials by generating population-based epitope maps, and also designed such maps using a large cohort of 372 naturally infected individuals. We used these maps to address several questions: (1) Are vaccine-induced responses randomly distributed across vaccine inserts, or do they cluster into immunodominant epitope hotspots? (2) Are the immunodominance patterns observed for these two vaccines in three vaccine trials different from one another? (3) Do vaccine-induced hotspots overlap with epitope hotspots induced by chronic natural infection with HIV-1? (4) Do immunodominant hotspots target evolutionarily conserved regions of the HIV genome? (5) Can epitope prediction methods be used to identify these hotspots? We found that vaccine responses clustered into epitope hotspots in all three vaccine trials and some of these hotspots were not observed in chronic natural infection. We also found significant differences between the immunodominance patterns generated in each trial, even comparing two trials that tested the same vaccine in different populations. Some of the vaccine-induced immunodominant hotspots were located in highly variable regions of the HIV genome, and this was more evident for the MRKAd-5 HIV vaccine. Finally, we found that epitope prediction methods can partially predict the location of vaccine-induced epitope hotspots. Our findings have implications for vaccine design and suggest a framework by which different vaccine candidates can be compared in early phases of evaluation.

## Author Summary

The HIV epidemic is a major global health challenge leading to more than 1.8 million deaths annually, and despite significant efforts, the search for an efficacious and safe vaccine continues. Several candidate vaccines were designed to elicit CD8+ T-cell responses and were based on using recombinant Adenovirus serotype 5 (rAd-5) vector that expresses HIV-derived antigens. While none of these vaccines had protective effects, they provide an opportunity to study vaccine-induced T-cell responses on a population level. Here, we analyze data from the three largest epitope mapping studies performed in three clinical trials testing two rAd-5 vaccines. We find that vaccine-induced responses tend to cluster in “epitope hotspots” and that these hotspots are different for each vaccine and more surprisingly in two different vaccine trials testing the same vaccine. We also compared vaccine-induced hotspots to those elicited by natural infection and found that some of the vaccine-induced hotspots are not observed in natural infection. Finally, we show that epitope prediction methods can be useful for predicting vaccine induced hotspots based on participants HLA alleles.

## Introduction

The HIV epidemic is a major global health challenge leading to more than 1.8 million deaths annually, and despite significant efforts the search for an efficacious and safe vaccine continues. Many different formulations of candidate HIV vaccines have been proposed and tested in recent years [1]. One of the leading approaches in this field focuses on vaccines that are primarily designed to elicit CD8^+^ T-cell responses that have been shown to be critical for controlling HIV infection [1]–[5]. These vaccines are comprised of vectored immunogens that use a modified virus (e.g. adenovirus or poxvirus) from which specific HIV genes are expressed. While several adenovirus types are currently being studied including rAd-35 and rAd-26 [6]–[8], only rAd-5 based HIV vaccines have been extensively tested to date. rAd-5 was chosen as a vaccine vector because previous work showed that it was both safe and highly immunogenic, eliciting vaccine-induced T-cell responses in 77% of the vaccinees [1], [9].

In the current study, we analyze epitope mapping data from two candidate rAd-5 HIV immunogens that were tested in human clinical trials. The MRKAd-5 HIV-1 gag/pol/nef vaccine was a multivalent rAd-5 vaccine that contained clade B gag/pol/nef HIV inserts and was tested in both a phase I trial (Merck16) [10] and a phase IIb trial (HVTN 502/Step) [11]. The VRC-HIVAD014-00VP was a multiclade, multivalent recombinant rAd-5 vaccine that contained a clade B gag-pol insert as well as envelope inserts from the three major HIV clades (A, B and C), and was tested in a phase I trial (HVTN 054) [12].

The HVTN 502/Step phase IIb trial was halted after an interim analysis showed that the tested vaccine did not reduce the rate of HIV-1 incidence nor reduce plasma viremia after infection [9], [11]. Considerable work has been conducted to identify potential reasons for the vaccine's failure. Preliminary analysis suggested an interaction between Ad-5 neutralizing antibody (nAb) titers and vaccine efficacy, but subsequent analyses failed to find a significant difference [13], [14]. A separate hypothesis was that the Merck vaccine, while highly immunogenic, induced only narrow responses generating a median of ≤1 T-cell response per participant [9].

Here, we sought to characterize the epitope responses generated by these two vaccines on a population level and used these epitope maps to address several questions: (1) Are vaccine-induced responses randomly distributed across vaccine inserts, or do they cluster into immunodominant epitope hotspots? (2) Are the immunodominance pattern observed in these three vaccine trials (two of which tested the same Merck vaccine product) different from each other? (3) Do vaccine-induced hotspots overlap with epitope hotspots induced by natural infection with HIV-1? (4) Do immunodominant hotspots target evolutionarily conserved regions of the HIV genome? (5) Can epitope prediction methods be used to identify these hotspots?

We found that vaccine-induced responses tended to cluster into immunodominant epitope hotspots in all three vaccine trials, and some of these hotspots were not observed in a chronic natural infection cohort. Comparing the hotspots induced in each trial, we found statistically significant differences between the patterns induced by Merck and VRC HIV vaccines, but also between the Merck16 phase I trial and the HVTN 502/Step trial that tested the same vaccine product in different populations. Some of the immunodominant hotspots targeted were from highly variable regions of the HIV genome, and this was most evident for the Merck vaccine. In addition, we showed that epitope prediction methods can partially predict the location of epitope hotspots and presented statistical tests for subsequently comparing these hotspots across different vaccine products and trials. Taken together our findings suggest that rAd-5 vector vaccines generate a clear immunodominance pattern on a population level, with many participants targeting similar areas. Specifically, they point to a small subset of regions within the HIV vaccine inserts that are highly immunogenic. These hotspots can be characterized experimentally with a relatively small number of participants (n<100), and, where there is knowledge about the importance of the hotspots for potential vaccine protection, may be used as novel immunogenicity endpoints for comparing candidate vaccine products and regimens [15] in phase I/II vaccine trials. Specifically, coupled with recent efforts to identify regions of the HIV genome to which responses are protective and non-protective [16]–[18], the identification of vaccine-induced epitope hotspots, may allow scoring different vaccine candidates based on their ability to generate responses to these protective regions. Furthermore, hotspots may potentially be used to define novel population-based biomarkers for assessment as immunological correlates of risk and protection in phase IIb/III efficacy trials [19], [20].

## Results

### Generating population-based epitope maps

To assess vaccine immunogenicity, we analyzed epitope mapping data from 177 vaccine recipients from three HIV-1 rAd-5 T-cell based vaccine clinical trials: Merck16, HVTN 054 and HVTN 502/Step, and compared the response patterns of each trial to those of 372 persons with chronic HIV infection (**Table 1**) [3]; constituting the three largest vaccine-induced epitope mapping studies to date. Epitope mapping was performed using IFN- γ ELISpot assays with sets of overlapping peptides, as detailed in **Table 1**. Responses were mapped down to the level of a single reactive K-mer peptide (K = 9–22). Specifically, high-resolution mapping was performed using 9 mers in both Merck16 and HVTN 054, 15 mers in HVTN 502/Step, and 15–20 mers in the natural infection cohort.

**Table 1:** Clinical datasets used in this study.

Trial	Phase	Immunogen	N mapped	N positive	Median number of responses	Mapping Strategy
**Step (502)**	IIb	Merck rAd-5-gag/pol/nef	71	52	3	Vaccine matched 15 mers (11 overlap)
**HVTN 054**	I	VRC-rAd-5 gag/pol/envA/B/C	34	32	3	conB 15 mers (11 overlap), additional optimal mapping
**Merck 16**	I	Merck rAd-5-gag/pol/nef	72	50	3	Merck matched 9 mers
**Natural Infection**	n/a	Full proteome mapping	372	372	19	conB 15–20 mers (10 overlap)

**Figure 1:**
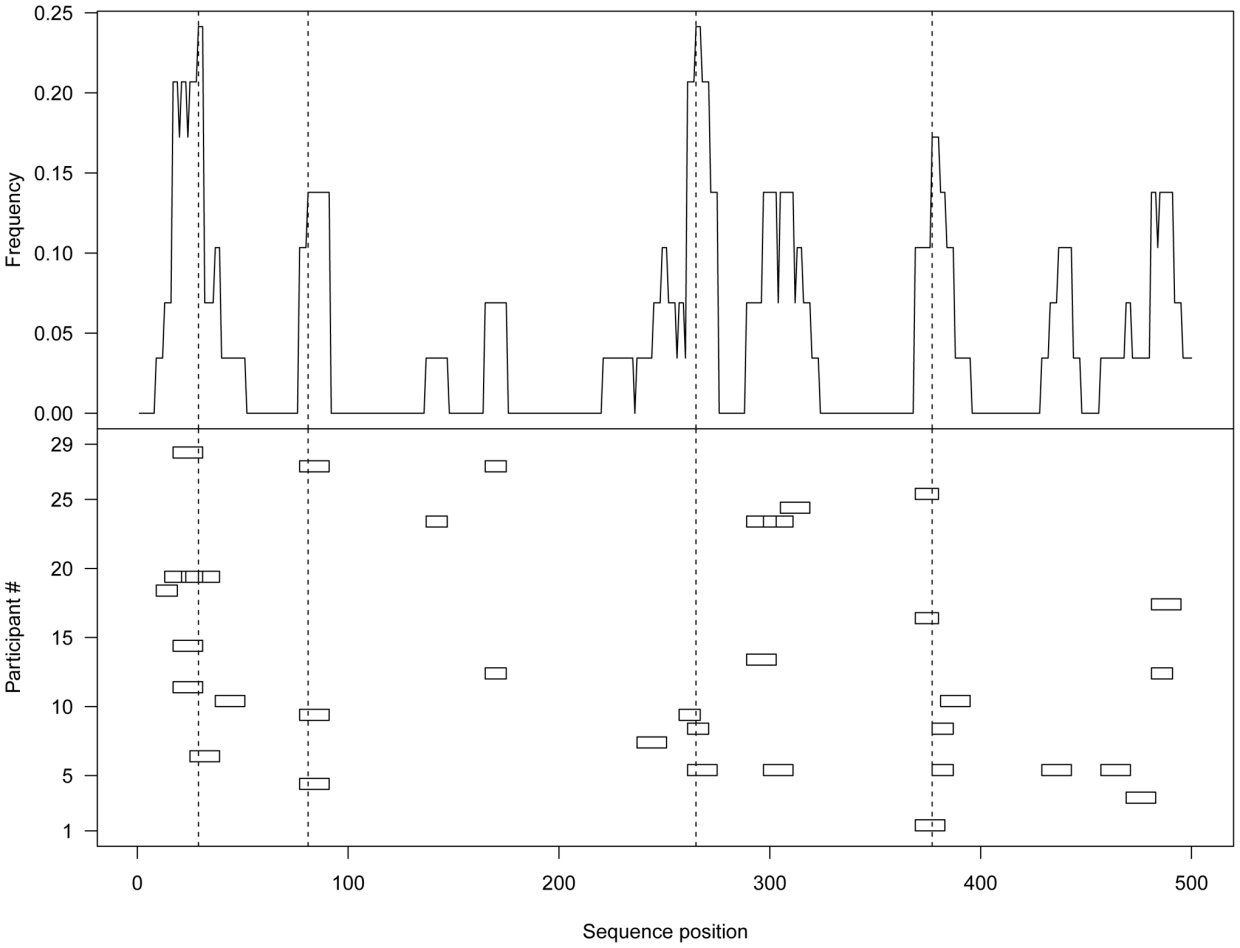
Generating population-based epitope maps. Epitope mapping data from 72 individuals from the HVTN 502/Step trial were obtained using and IFN-γ ELISPOT assay with sets of overlapping 15-mer peptides that span the HIV-1 Gag Step vaccine insert. Responses of the 29 individuals that had at least a single epitope response to the gag insert are plotted on the bottom part of the figure. Each row represents a single participant. Each box represents a single response. Responses to overlapping positions are marked by overlapping boxes. These responses are then summed up to create the population-based map shown on the top part of the figure, in which frequencies of detection for each site along Gag are shown for this cohort. Consecutive responses made by a single individual were consolidated into a single response at the intersection of the two adjacent peptides.

Our goal was to characterize HIV-1 vaccine-induced CD8+ T-cell responses on a population level. We therefore used the epitope mapping data of each cohort to create population-based epitope maps by tallying the number of responses that were observed for each position along a given HIV-1 protein. Counts were then normalized to provide population-based detection frequencies (**Figure 1**). Maps were generated using conservative estimates of the number of epitope responses for each individual by considering consecutive positive K-mers as a single epitope response (see Materials and Methods). These maps were also used to compare immune response patterns between recipients of the different vaccines as well as to persons with natural infection with HIV-1, focusing on the location the most immunodominant hotspots in each trial.

The Merck16 and HVTN 502/Step trials used an identical rAd-5 gag/pol/nef vaccine developed by Merck Laboratories. The HVTN 054 trial administered a rAd-5 gag-pol/envA/envB/envC vaccine developed by the NIH Vaccine Research Center. While the two vaccines contained different immunogens, their gag and pol inserts were both clade-B isolates that were extremely similar to one another [10], [12], and both were based on an rAd-5 backbone.

### Vaccine-induced epitope responses cluster in immunodominant epitope hotspots

**Figure 2:**
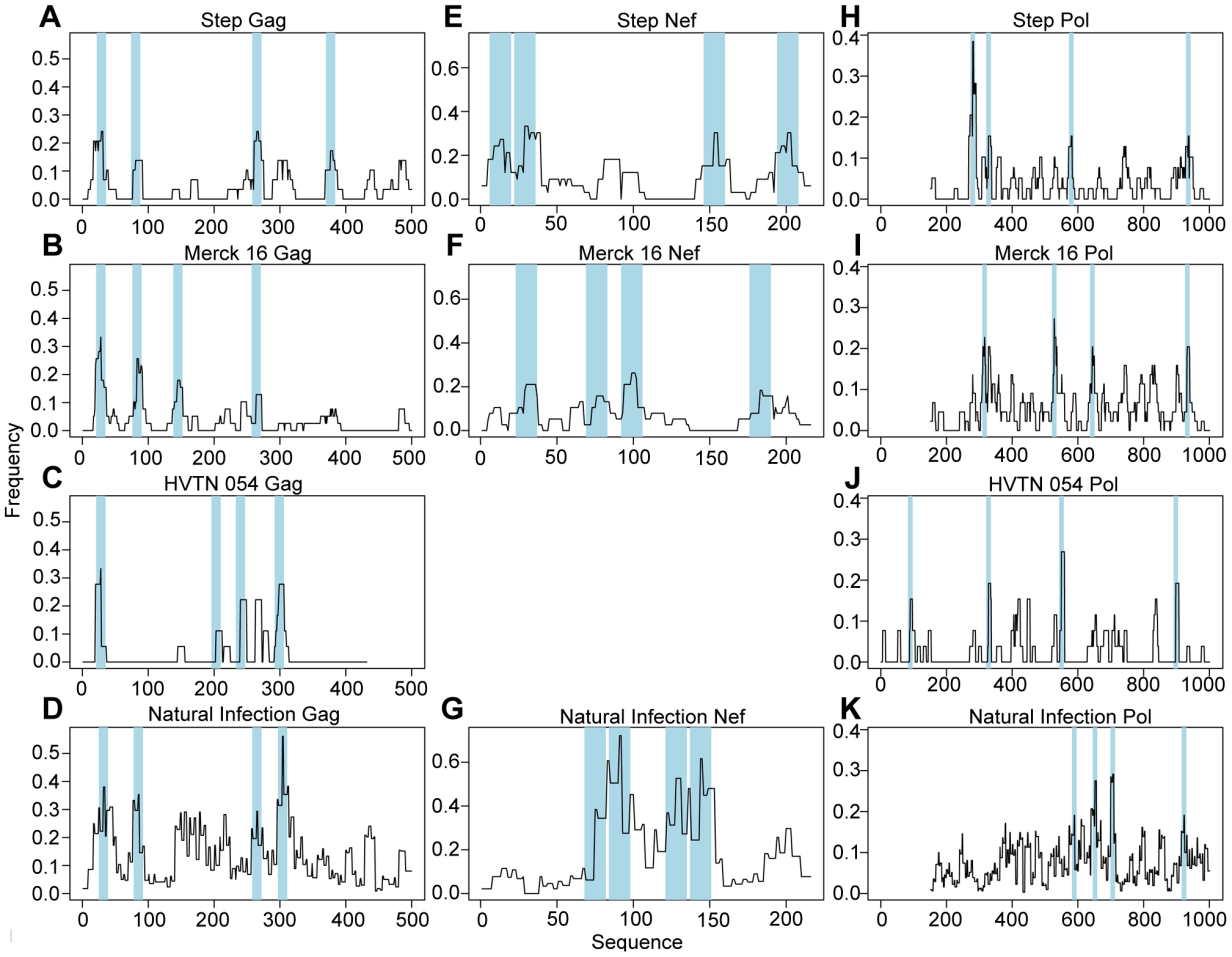
Different vaccines induce different immunodominant hotspots. Population-based epitope maps for each protein are presented for HVTN/Step 502, Merck16, HVTN 054 and the Natural infection cohort. Shaded regions mark the four most frequent immunodominant hotspots in the HVTN 502/Step maps. (a–d) Population-based epitope map of Gag. (e–g) Population-based epitope maps of Nef. (h–k) Population-based epitope maps of Pol.

An epitope hotspot is typically defined as a public immunodominant region that contains several epitopes that are presented by different HLA alleles and is targeted by many individuals. In this study we defined hotspots statistically as sets of contiguous sites along a protein that were targeted more frequently than under the null hypothesis of equal targeting frequencies for all sites. Using permutation tests on the location of epitopes for each participant, we found that epitope responses in all three vaccine trials clustered in immunodominant hotspots (p-values ranging from 0.0001–0.07 in all vaccine inserts) (**Table 2**). Most vaccine inserts contained at least four statistically significant hotspots (**Figure 2**); these were also observed among naturally infected persons [3] (**Figure 2**).

**Table 2:** Epitope hotspots observed in each vaccine trial.

Trial	Protein	Hotspot sequence	Start position	End position	Response rate	Restricting HLAs	P-value
Step	gag	GGELDKWEKIRLRPGGKKKYKLKHIVWASR	10	39	0.310345	A*0301, A*2402, B*3905, B*5501	0.0004
Step	gag	QIGWMTNNPPIPVGEIYKRWIILGLNKIVR	246	275	0.344828	A*0201, A*1101, A*2402, A*3101, B*0801, B*2705, B*3501, B*3913	0.0460
Step	gag	DRFYKTLRAEQASQEVKNWMTE	298	319	0.206897	A_0201, B_3913, B_4403	0.3400
Step	gag	VTNSATIMMQRGNFRNQRKTVK	370	391	0.310345	A*1101, B*2705, B*3905	0.0400
Step	nef	AGKWSKRSVPGWSTVRERMRRAEPAADRVRRTEPAAVGVGAVSRDLEKHGAITSSNTA	2	59	0.575758	A*2402, A*3101, A*3301, A*6801, B*0702, B*1504, B*2705, B*3701, B*3801, B*3913, B*5101, B*5131, B*5301	0.0740
Step	nef	FPVRPQVPLRPMTYKGAVDLSHFLKEKGGL	78	107	0.363636	A*1101, B*0702, B*3543, B*4402, B*4901	<0.0001
Step	nef	GIRFPLTFGWCFKLVPVEPEKVEEAN	142	167	0.515152	A*0201, A*0301, B*1801, B*3517, B*3905, B*4403	0.0100
Step	nef	MSQHGIEDPEKEVLEWRFDSKLAFHHVARELHPEYYKDC	178	216	0.484848	A*0201, A*2402, A*3101, B*2705, B*3905, B*3913, B*5101, B*5301	0.0001
Step	pol	FSVPLDEDFRKYTAFTIPSINNETPGIRYQ	118	147	0.666667	A*0201, A*0211, A*2402, A*2905, A*3301, B*0702, B*5101, B*5131	<0.0001
Step	pol	RKQNPDIVIYQYMAALYV	174	191	0.25641	A*0201, A*0206, B*3501, B*3508	0.2100
Step	pol	FVNTPPLVKLWYQLEKEP	418	435	0.230769	A*1101, A*2905, A*9209, B*3501, B*3543	0.0400
Step	pol	GGYSAGERIVDIIATDIQTKELQKQITKIQNFRVYYRDSRNPLWKG	754	799	0.25641	A*0201, A*0205, A*0301, A*1101, A*2402, A*3002, B*4402, B*4403, B*5101	0.0040
HVTN 054	envA	VYYGVPVWK	37	45	0.631579	A*2301, A*2901	<0.0001
HVTN 054	envA	DAETTLFCASDAKAYDTEVHNVWETHACVPTD	46	77	0.210526	A*0201, B*3501, B*3503, B*3508	0.2600
HVTN 054	envA	WGIKQLQARVLAVE	520	541	0.263158	A*3301, B*0801, B*1402, B*3501	0.0010
HVTN 054	gag	LRPGGKKKYKLKHIVW	21	36	0.333333	A*2402	0.0070
HVTN 054	gag	PGQMREPRGSDIAGTT	225	240	0.222222	B*4402, B*5701, B*5801	0.0020
HVTN 054	gag	STLQEQIGW	241	249	0.222222		0.9800
HVTN 054	gag	FRDYVDRFYKTLRAEQASQEV	293	313	0.277778	A*0201, A*0205, B*1402, B*5101	0.0002
HVTN 054	pol	QNPDIVIYQY	327	336	0.192308	B*3501, B*3508	0.0330
HVTN 054	pol	IQKETWEAWWTEYW	546	559	0.269231	A*2301, B*4403, B*4405, B*4901	0.0040
HVTN 054	pol	AGIKQEFGIPYNPQSQGVIESMNKELKKIIGQVRDQAEHLKTAVQMAVFIHNF	846	898	0.192308	A*2901, B*1503, B*2705	0.0010
HVTN 054	pol	KRKGGIGGY	899	907	0.192308		0.0570
Merck16	gag	RLRPGGKKKYKLKHIVWA	20	37	0.384615	A*0201, A*0301, A*2402, A*2403, B*1501, B*3801, B*3901	<0.0001
Merck16	gag	LYNTVATLYCVHQKIDVKD	78	96	0.333333	A*0201, A*0301, A*1101, A*3002, B*3501	<0.0001
Merck16	gag	LQGQMVHQAISPRTL	138	152	0.179487	A*0201, B*1302, B*3901, B*5101	0.0009
Merck16	gag	MTNNPPIPVGEIYKR	250	264	0.128205	A*0301, A*2402, A*3303	0.3300
Merck16	nef	MRRAEPAADRVRRTEPAA	20	37	0.236842	A*0201, B*0702, B*1402, B*1501, B*3901	0.0190
Merck16	nef	EEVGFPVRPQVPLRP	74	88	0.157895	A*1101, B*5101	0.3748
Merck16	nef	AVDLSHFLKEKGGL	94	107	0.263158	A*0301, A*1101, A*3002, B*0801, B*3801	0.1030
Merck16	nef	MSQHGIEDPEKEVL	178	191	0.184211	B*4001, B*5301	0.1890
Merck16	pol	GWKGSPAIFQSSMTKILEPF	154	173	0.227273	A*0201, A*0301, A*1101, B*0702, B*1520, B*3501	<0.0001
Merck16	pol	LTEAVQKITTESIVIWGKTPKFKLPIQKETWETWWTEYW	370	408	0.272727	A*2402, A*2403, B*1301, B*1302, B*3801, B*4402, B*4403, B*5101, B*5801	0.0054
Merck16	pol	QAIYLALQDSGLEVNIVTASQYALGII	482	508	0.25	A*0101, A*2402, A*2403, A*2902, B*1401, B*3503, B*3801, B*5101, B*7801	0.0002
Merck16	pol	IQNFRVYYRDSRNPL	782	796	0.227273	A*2402, B*4402	0.0002

The four dominant hotspots for each protein are presented. Restricting HLAs were imputed using HLA binding predictors.

### Different vaccines induce different immunodominant hotspots

We next sought to compare the location of these epitope hotspots among the groups by developing a statistical test that was based on the targeting frequency of the most dominant hotspot for each cohort (see Materials and Methods for “targeting frequency” calculation). Using a permutation test, we ascertained if the maximal difference in targeting frequency was higher than expected under equal frequencies. We found significant differences in Pol between HVTN 502/Step and HVTN 054 (p = 0.045, **Figure 2h,j**) and between HVTN 502/Step and Merck16 (p = 0.0041, **Figure 2h,i**). We also used a permutation test to ascertain if the sum of differences in targeting frequencies was higher than expected under equal frequencies. We found significant differences in Gag and Pol between HVTN 502/Step and HVTN 054 (Gag: p = 0.045, **Figure 2a,c**; Pol p = 0.0007, **Figure 2h,j**), and in Nef and Pol between HVTN 502/Step and Merck16 (Nef p = 0.0028, **Figure 2e,f**; Pol: p = 0.05, **Figure 2h,i**).

**Figure 3:**
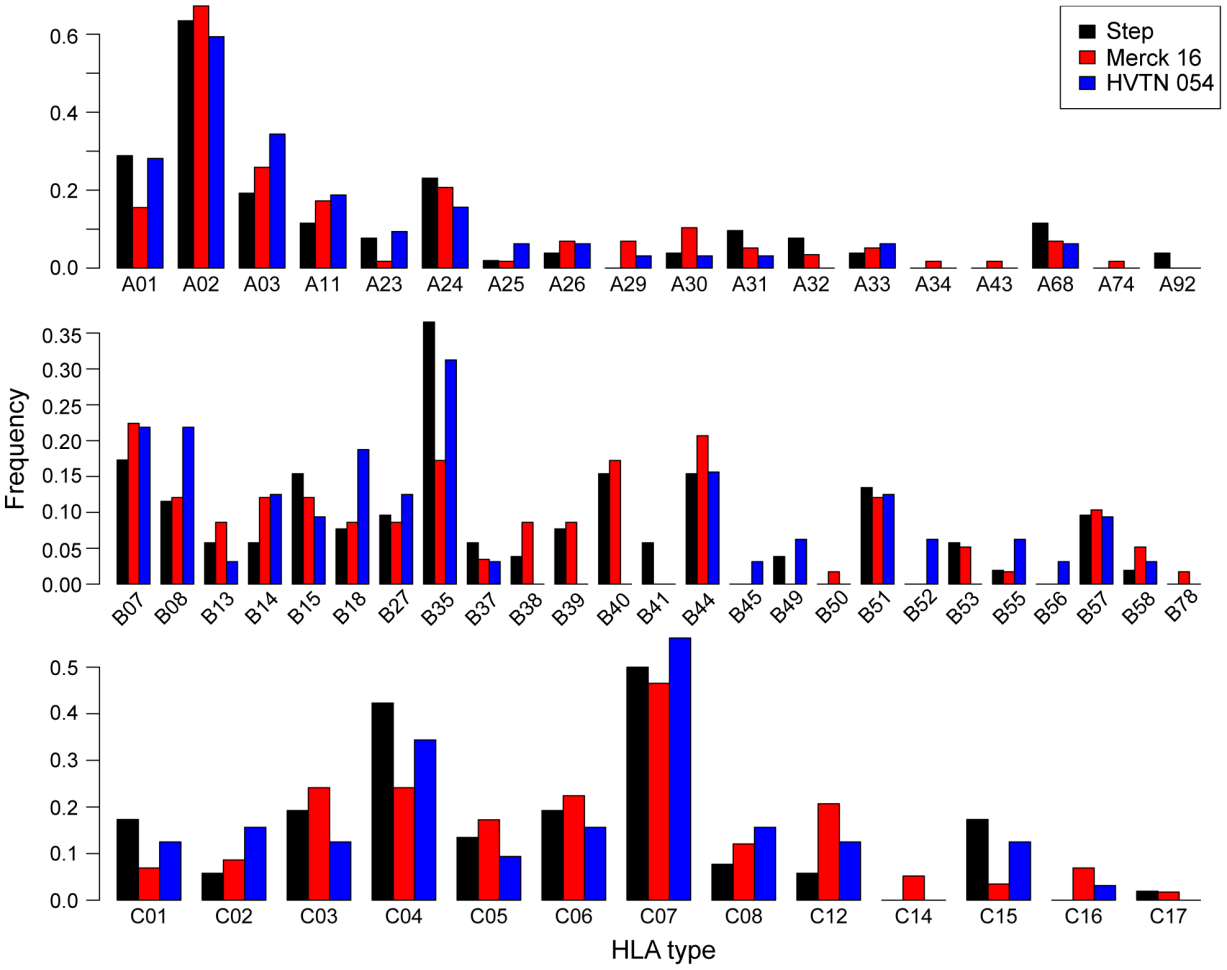
HLA distributions of participants in the three clinical trials. Differences between tests were not significant (p = 0.1825, Fisher's exact test).

We then compared the frequency of HLA alleles in these cohorts, which may bias responses towards specific epitope hotspots targeted by different HLA alleles. In comparing these distributions we found no evidence for significant differences between the three trials (Fisher's exact test p = 0.43, **Table 3** **,** **Figure 3**). To further address this hypothesis, we used an HLA matching strategy to identify HLA strata that are comparable between HVTN Step/502 and Merck16 (see methods for details). We then recomputed the two statistical tests for hotspot differences described above accounting for HLA strata. After correcting for HLA we found no evidence for statistical differences in hotspot locations in Gag, but the differences in Nef and Pol remained significant (**Table 4**). This suggests that it is unlikely that all of the differences in the immunodominant hotspots observed in these two trials are a result of differences in the HLA distributions of trial participants.

**Table 3:** Comparisons of HLA distributions of the different vaccine trials.

Clinical trials compared	p-value
HVTN Step vs. HVTN 054	0.624
Merck16 vs. HVTN 054	0.3961
Merck16 vs. HVTN Step	0.2811

Distributions were compared using Fisher's exact test.

**Table 4:** P-values for the max and sum tests of differences between population hotspots maps of HVTN Step and Merck16 with and without HLA stratification.

Protein	Original analysis		HLA stratification	
	Max test	Sum Test	Max test	Sum Test
	p-values	p-values	p-values	p-values
Gag	0.457	0.079	0.801	0.429
Pol	0.004	0.050	0.004	0.086
Nef	0.078	0.003	0.082	0.014

### Some vaccine-induced epitope hotspots target regions that are not frequently targeted in natural infection

We compared the vaccine-induced immunodominant hotspots to those elicited from natural infection to determine how similar the T-cell responses are between these different populations. It has been previously shown that T-cell responses during natural infection are of higher magnitude and breadth than those resulting from HIV vaccination [3], [4], [21], [22], and that on a population level, almost all regions of a given HIV-1 protein are targeted by a T-cell epitopes [3]. We therefore asked two questions: (1) what is the correlation between the vaccine-induced and natural infection-induced epitope maps; and (2) are there epitope hotspots that are targeted following vaccination that are not frequently targeted during natural infection? We first computed the Spearman correlation between vaccine-induced maps and natural infection maps. We found that natural infection response patterns to Gag were positively correlated with the patterns induced by the vaccines in HVTN 502/Step (r = 0.42, p<10^−5^), Merck 16 (r = 0.42, p<10^−5^) and HVTN 054 (r = 0.40, p<10^−5^) (**Figure 2a–d**). However, natural infection response patterns to Nef were not significantly correlated with response patterns for HVTN 502/Step (r = −0.07, p = 0.29) and Merck16 (r = 0.10, p = 0.15) (**Figure 2 e–g**). Similarly, no or very weak correlations were found between the natural infection responses to Pol and those elicited by HVTN 502/Step (r = −0.03, p = 0.34), Merck16 (r = −0.07, p = 0.048), or HVTN 054 (r = 0.069, p = 0.042) (**Figure 2h–k**). We then asked if any of the vaccine-induced hotspots targeted areas that were not frequently targeted in natural infection, i.e. if they introduced any novel immunodominant hotspots. We identified several hotspots in both Gag (**Figure 2a,d**) and Nef (**Figure 2e,g**) that were targeted by HVTN 502/Step vaccine recipients, but not targeted in chronic natural infection.

### Some of the vaccine-induced immunodominant hotspots target non-conserved sites

**Figure 4:**
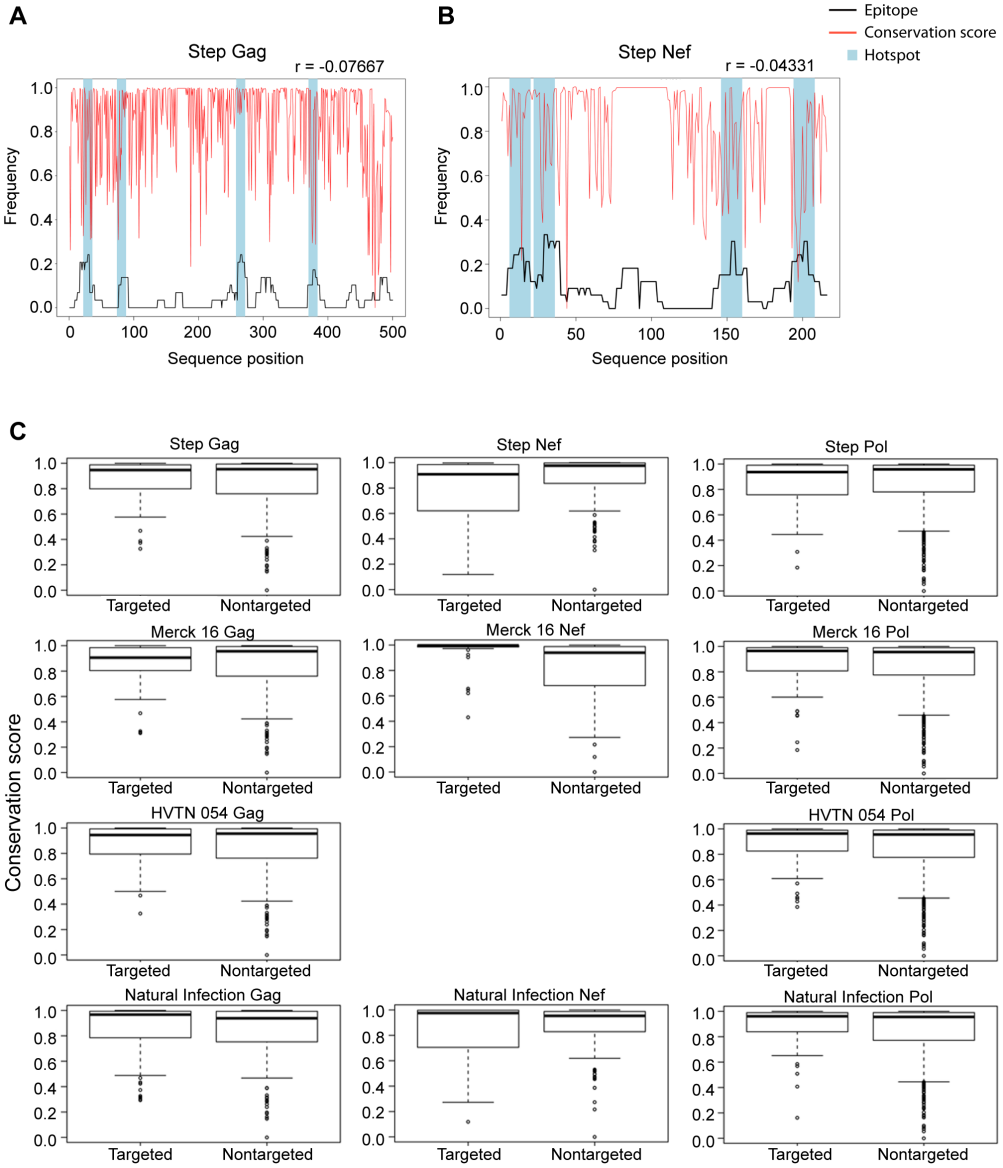
Some vaccine-induced hotspots target highly variable regions of HIV. Population epitope maps of HVTN 502/Step for Gag (a) and Nef (b) overlaid with conservation scores (red line). Conservation scores were scaled to the range of 0–1, where lower scores correspond to more variable sites. The four immunodominant hotspots are shaded in blue. (c) Boxplots comparing the conservation patterns of hotspots vs. non-targeted sites along each protein are presented. Targeted hotspots were defined as areas targeted by more than 15% of individuals in a trial vs. all other sites along the protein. P-values reported are based on the Wilcoxon rank sum test. Medians are represented by black lines, and the bottom and top of the box denotes the 25^th^ and 75^th^ percentiles, respectively. The whiskers extend to the most extreme data point, which is no more than 1.5 times the interquartile range from the box and outliers are marked by circles.

In order to assess the relationship between targeted hotpots and evolutionary conservation, we computed HLA targeting efficiency scores for each population-based epitope map. The HLA targeting efficiency score is defined by the Spearman correlation coefficient between binding scores and conservation scores for amino acids along a given protein [23]. A positive score indicates preferential binding to conserved sites along the protein and a negative score indicates preferential binding to variable regions. In a previous study, we found that the HLA alleles preferentially targeted the conserved regions of pathogens and self proteins [23]. In accordance with those findings, the HLA targeting efficiency scores of the natural infection epitope maps were positive for both Gag and Nef (**Table 5**). Surprisingly, some of the efficiency scores of the vaccine induced maps were negative, indicating preferential binding to variable regions. For example, the efficiency scores of Gag were negative in all vaccine trials, but were positive in the natural infection cohort. To further characterize this phenomenon, we overlaid the conservation scores of each site of each protein over the population-based epitope maps. Indeed, we found that most epitope hotspots in both Gag and Nef contained highly variable sites (**Figure 4a–b**). We then compared the conservation scores of hotpots vs. other sites for each of these epitope maps (**Figure 4c**). For Merck16, the targeted sites of Nef had significantly higher conservation scores than non-targeted, but an opposite trend was observed for HVTN 502/Step.

**Table 5:** Epitope hotspots and their relationship to evolutionary conservation as measured by the HLA targeting efficiency scores.

Trial	Protein	Efficiency score	p-value	97.5% CI	
**Step (502)**	Gag	−0.076	0.113	−0.150	0.015
	Nef	−0.043	0.75	−0.193	0.163
	Pol	0.005	0.84	−0.034	0.041
**HVTN 054**	Gag	−0.046	0.015	−0.095	−0.004
	Pol	0.007	0.65	−0.015	0.029
**Merck 16**	Gag	−0.012	0.75	−0.077	0.053
	Nef	0.35	0.0002	0.155	0.441
	Pol	−0.015	0.67	−0.042	0.034
**Natural Infection**	Gag	0.064	<0.0001	0.035	0.092
	Nef	0.083	0.0002	0.039	0.124
	Pol	−0.075	<0.0001	−0.090	−0.050

Scores can range from −1 to 1. Positive scores indicate that epitopes tend to be in the more conserved regions of the targeted protein, while negative scores indicate targeting of the more variable sites along the insert. P-values were computed using the bootstrap procedure, and denote the significance of the correlation between epitope hotspot location and evolutionary conservation.

### Epitope prediction methods can be used to identify vaccine-induced immunodominant hotspots

Motivated by the need to improve sampling designs for expensive immunological endpoint experiments, which require both large quantities of PBMCs and numerous procedures, we sought to determine if HLA binding predictors can be used to identify epitope hotspots. Several recent benchmarks have shown that HLA binding predictors are highly accurate and can also be used to predict binding to HLA alleles that have not been experimentally characterized, building upon other alleles for which experimental data are available [24], [25].

**Figure 5:**
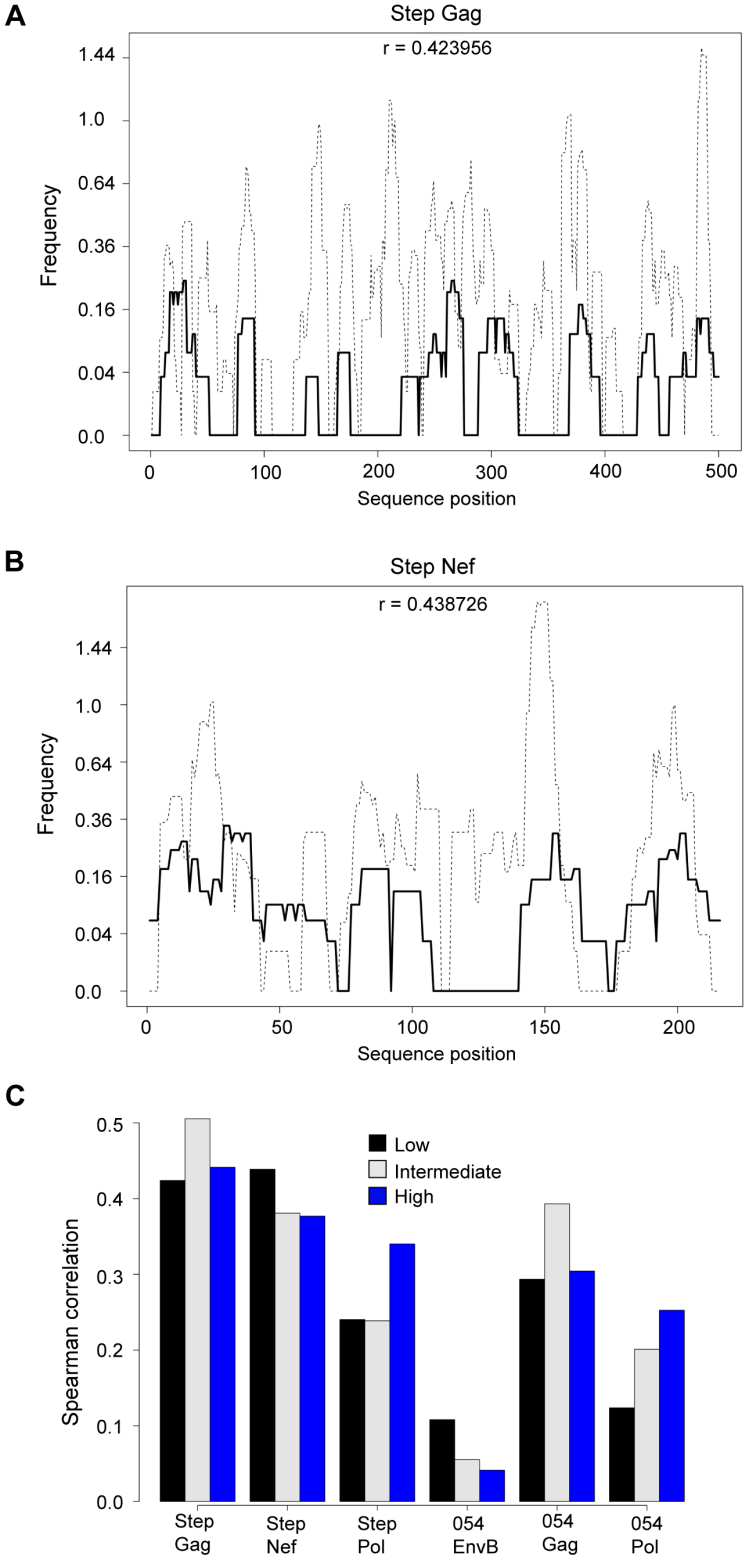
HLA binding prediction algorithms can be used to identify vaccine-induced immunodominant hotspots. A comparison of the measured (black) vs. predicted (dashed) population-based epitope maps in HVTN 502/Step. Predictions were computed using the HLA alleles of trial participants and were weighted by HLA frequencies in this cohort. (a) Predicted vs. measured map for Gag. (b) Predicted vs. measured map for Nef. (c) Spearman correlation coefficients between measured and predicted epitope maps. Predicted maps were generated using three different IC50 thresholds on predicted binders: low (50 nM), intermediate (150 nM) and high (500 nM). Different thresholds yield higher correlations for different proteins: for all proteins but Pol, lower thresholds yield higher correlations, suggesting that the responses detected by the experimental assay are focused on the most immunogenic, high-affinity HLA binders. Since predicted population-based maps were obtained using predicted 9-mers, peaks in these maps are by definition more narrow than the experimentally measured maps in which 15 mers were used for epitope mapping.

Here we developed a population-based approach in which we pool predictions for all HLA alleles into one prediction map. The approach is based on the observation of immunological hotspots targeted by many different HLA alleles. Population maps were generated by tallying the number of predicted 9 mers that straddled each position along a vaccine insert given the HLA alleles of the trial participants. Predicted binders were defined as 9 mers for which the predicted IC50 value was below a threshold δ (δ = 50 nM, 150 nM, and 500 nM). We compared the measured population-based epitope maps to the prediction maps (**Figure 5a–b**).

We found that the predicted maps were significantly correlated to the measured maps for Gag, Pol and Nef but less so for Env (r values ranging from 0.07 for Env to 0.5 for Gag, **Figure 5c**). In almost all cases the immunodominant measured peaks were identified in the prediction maps. However, predicted maps contain several additional peaks that were not detected experimentally. This is not surprising due to the fact that the predicted maps are based solely on HLA binding, which is only one determinant of immunodominance, and does not take into account proteasomal processing, epitope half-life, TAP transport and expression on cell surface, TCR avidity, and other intrinsic properties of individual T cells.

## Discussion

In this study, we analyzed epitope mapping data from three independent HIV vaccine clinical trials that evaluated two distinct rAd5-based vaccines, and made comparisons to a large natural infection cohort. Unlike responses in natural infection, which were measured using consensus clade B peptides and based on PBMC samples from varying timepoints following infection, vaccine-induced responses were measured using vaccine-matched peptides for HVTN Step and Merck16 and clade-B peptides (highly similar to the vaccine insert) for HVTN 054; in all vaccine trials, responses were assayed at a fixed timepoint following vaccination. These factors significantly increase our confidence in the vaccine-induced epitope maps, since responses were not lost due to mismatches between the tested peptides and the autologous epitope or to viral escape via mutations in and around epitopes. We presented statistical tests for identifying and comparing epitope hotspots across different vaccine products and trials. We found that both vaccines induced highly significant epitope hotspots. To our knowledge, this is the first study to utilize epitope hotspots for the comparison of different vaccine trials and products by the immunodominance patterns that they induce.

We found that the immunodominance hierarchies generated by each vaccine were distinct from one another. The differences between the two Merck vaccine trials are surprising, since they used the same vaccine. We further showed that some of the vaccine-induced hotspots were not frequently observed in a large cohort of chronic naturally infected individuals.

While we found no evidence for significant differences in the HLA frequencies between the three trials (**Table 3**), and found that some of the differences between the immunodominance patterns between HVTN 502/Step and Merck16 were still significant after HLA stratification (Nef and Pol, **Table 4**), there are several other potential explanations for the differences in the immunodominance patterns that were observed here. First, there is lack of power to detect the full landscape of immunodominant responses in each trial. This is partially supported by the finding that correlations between predicted and measured epitope maps were stronger for some vaccine inserts when considering only high-affinity predicted binders (**Figure 5c**), and also by differences found between HVTN 502/Step and Merck16. We note however that the data analyzed here included the three largest T-cell epitope mapping studies performed in HIV-1 vaccine trials, and as such are the best existing data currently available. A second potential explanation is that slight changes in the immunogen can lead to large differences in the immunodominance patterns that they induce. While both the Merck vaccine and the VRC vaccines were based on a rAd-5 backbone, they had several important differences: (1) the VRC product included a Gag-Pol fusion in a single insert and the Merck product contained a separate vector for Gag and Pol; (2) the VRC vaccine also included Env inserts which could have provided epitope competition for MHC binding; and (3) vector design – the GenVec rAd5 used in HVTN 054 was E4 and E3 partially deleted, and these regions were not deleted in the Merck rAd5 vector. E4 is required to produce Ad structural proteins such as hexon, which can activate cell signaling processes that can affect the proteasome and the “inflammasome.”

Accordingly, the most immunodominant hotspot in HVTN 502/Step (targeted by more than 40% of participants) was not observed in HVTN 054. A similar effect, albeit for antibodies, was recently reported for the RV144 vaccine regimen in which the replacement of the C-terminus of gp120 with a gD tag modified the antibody immunogenicity pattern induced by this immunogen [26]. Another potential explanation is differences in the epitope mapping strategies used in each study. While Merck16 and HVTN 502/Step were mapped using vaccine matched peptide sets, HVTN 054 and the natural infection cohort were mapped using consensus B peptides. Merck16 was mapped with vaccine matched 9 mers in the Merck laboratories, while HVTN 502/Step and HVTN 054 were mapped with 15 mers in the HVTN laboratory, and the natural infection cohort was mapped with 15–20 mers in a third laboratory. The differences observed between epitope hotspots in natural infection to those induced by vaccination could also be due to changes in the immunodominance patterns between acute and chronic infection [5]. Since some of the vaccine-induced hotspots were in more variable regions of the HIV genome, they may not be chronic immunodominant hotspots due to T-cell escape. It may therefore be important to compare responses in acute infection to vaccine-induced responses. While it is impossible to tease out which of these factors (or combination thereof) were responsible for these differences, we believe they highlight the importance of conducting additional studies to unravel the underlying factors that influence the immunodominance patterns in a vaccine setting.

We note that the epitope maps described in this paper were based on epitope prevalence and not on the actual magnitudes of the T-cell responses as measured by ELISPOT. Therefore, some of these may be hotspots of low-magnitude responses. We found that the average ELISPOT response measured in Step was 432 SFC/M. Furthermore, the average response of the two most prevalent peaks in HVTN 502/Step Gag (**Table 2**) was 534 SFC/M and 593 SFC/M, accordingly. This suggests that prevalent hotspots were also magnitude hotspots.

An analysis of the evolutionary conservation of vaccine-induced hotspots showed that some hotspots were directed against highly variable sites in the HIV genome, in which the virus can readily tolerate a variety of mutations that may allow escape from immune recognition (see also [27]). A consequence of this finding is that breakthrough infections in these trials are likely to lead to early post-acquisition mutations which do not incur significant fitness cost. Indeed, sieve analysis of breakthrough infections in the HVTN Step trial found evidence for T-cell sieve effects in both Gag and Nef [28].

Finally, we showed that epitope prediction methods can be used to predict the location of epitope hotspots in vaccine trials. Importantly, predicted epitope maps tended to overshoot – predicting additional hotspots that were not seen in the empirical epitope mapping, and only rarely missed a measured hotspot. This suggests two uses of epitope prediction methods for potentially improving epitope mapping protocols in clinical trials. First, given the HLA frequencies of the target population, prediction models can be used to identity epitope hotspots that can contribute to the scoring and comparing of candidate vaccines as outlined below. Second, binding predictors may potentially be incorporated into the epitope mapping protocol itself, allowing a more focused investigation of epitopes that is tailored for each individual based on their HLA alleles, thereby reducing the number of tests required for epitope mapping. However, while the correlations between predicted and experimental epitope maps were encouraging, additional research and validation studies are needed to develop new epitope mapping algorithms that combine epitope predictions with direct epitope measurements; such algorithms should be shown to be at least as specific and sensitive as current epitope mapping protocols before they merit use.

Taken together, our findings demonstrate that vaccines can generate a clear immunodominance pattern on a population level. Specifically, they suggest that by fine-mapping the immune responses in early Phase I or Phase IIa trials one may obtain a complete set of the regions that are likely to be targeted by the specific vaccine candidate, and those regions can then be further analyzed in terms of their functional importance, evolutionary conservation and any other biological property of interest to determine if targeting these regions is likely to provide any functional effect on HIV acquisition or replication capacity. A recent report comparing T-cell responses to Gag in HIV controllers vs. non-controllers, excluding individuals with protective HLA alleles, found that while the breadth and magnitude of responses in both groups were comparable, responses in the controllers were more cross-reactive and of higher avidity than those in the non-controllers [17]. Another study identified peptides that had a “protective ratio” by comparing the viral loads of responders and non-responders to each peptide [16]. Similarly, Dinges et al. reported that T-cell responses were better predictors of HIV disease progression than HLA alleles [18]. These data point to the possibility of defining an importance function that can be used to weight different positions within a vaccine insert. Combining such a weighting function with experimental epitope mapping data can provide a powerful tool to assess and compare different candidate HIV vaccines in early stages of their development [15], [19], [20].

## Materials and Methods

### Clinical datasets

We analyzed data from three HIV-1 vaccine clinical trials that administered immuonogens based on a replication defective rAd-5 vaccine vector into which several HIV proteins were inserted. We also analyzed data from a natural infection HIV-1 cohort.

Merck16 – a phase I trial of the MRK rAd-5 HIV-1 gag/pol/nef vaccine developed by Merck Research Laboratories that enrolled 259 participants [10], [27]. The vaccine was a 1∶1∶1 mixture of rAd-5 constructs containing HIV-1 clade B *gag*, *pol*, and *nef* which were inserted into the E1 region of the rAd-5 backbone.

HVTN 054 – a phase I trial of an Ad5 Gag/Pol/EnvA/EnvB/EnvC vaccine developed by the Vaccine Research Center that enrolled 48 participants. The product contained a mixture of 3∶1∶1∶1 E1-, partially E3-, and E4- deleted rAd5 constructs expressing a *gag-pol* fusion gene from HIV-1 subtype B and *env* genes (from subtypes A, B, and C) from the E1 region of the rAd-5 backbone [12]. Epitope mapping was performed on samples obtained 4 or 12 weeks after vaccination.

HVTN 502/Step – a phase IIb trial of the MRK rAd-5 gag/pol/nef vaccine given at months 0, 1 and 6 that enrolled 3,000 participants. The trial was unblinded after an interim analysis found that vaccine recipients had a higher risk of infection as compared to placebo recipients [11]. Epitope mapping was performed on samples obtained 4 weeks after the second vaccination.

Natural infection cohort – 372 HIV-1 clade B chronically infected subjects were recruited from four hospitals in the Boston area and at the Queen Elizabeth Hospital in Barbados, as previously described [3]. Briefly, this cohort included predominantly chronically infected participants, some of which were undergoing anti-retroviral treatment.

All data analyzed in this study were de-identified, and the study was approved by the HVTN review committee.

### Epitope mapping

Epitope mapping was performed using a group testing approach [2], [29] in which T-cell responses are measured using an IFN-γ ELISPOT assay as previously described. Briefly, peptides representing Gag, Pol, Nef and Env were tested in pools, and peptides contained in positive pools were further tested individually. Responses to individual peptides were considered positive if they were at least threefold above the average of at least six negative control wells (containing the peptide diluent) and ≥50 spot forming cells (SFC)/10^6^ PBMC. Responses were first measured to master or matrix pools of overlapping 9 mer (Merck16), 15 mer (HVTN 054, HVTN 502/Step) or 15–20 mer (natural infection cohort) peptides, each containing 40–100 peptides that span the vaccine immunogens. Positive responses were then further tested using minipools containing 5–10 peptides. The reactive 9 mers/15 mers were identified by testing each individual 15 mer from all reactive pools.

Epitope mapping of Merck16 was performed by Merck Laboratories and included 72 participants. Responses were mapped using vaccine-matched 9 mer peptides that spanned all three immunogens with consecutive peptides overlapping by 5 amino acids. Positive responses were defined as responses that were over 50 SFC and three times higher than background responses, as relatively high backgrounds were observed in this study (see [27] for further details).

Epitope mapping of HVTN 054 was performed by the HVTN laboratory and included 29 participants. Responses were measured using 15 mer peptides that spanned a consensus HIV-1 clade B (conB) strain that closely resembles but does not match the vaccine strain. Consecutive peptides had an overlap of 11 amino acids. Positive pool responses were defined as responses that were over 50 SFC and were two times higher than background. Positive 15 mer responses were further de-convoluted to identify the optimal epitope, based on sample availability.

Epitope mapping of HVTN 502/Step was performed by the HVTN laboratory and included 71 participants. Responses were measured using vaccine matched 15 mer peptides with an overlap of 11 amino acids between each consecutive pair of peptides. Positive responses were defined as responses that were over 50 SFC and were three times higher than background.

Epitope mapping of the natural infection cohort (n = 372) was performed across the entire HIV-1 genome using conB overlapping 15–20 mers, with an overlap of 10 amino acids between adjacent peptides. Positive responses were defined as responses that were 4 times background levels and higher than 50 SFC.

### HLA typing

Four-digit HLA class I typing was performed on all trial participants for whom we had epitope mapping data. HLA typing was not available for the natural infection cohort. **Figure 3** presents the distributions of HLA class-I alleles for the participants within these trials, and includes a statistical comparison of these distributions.

### Generating population-based epitope maps

Population-based epitope maps were generated for each vaccine insert by tallying the number of reactive 15 mers that included a given site across all study participants. The frequency of response was calculated by dividing the number of responses at a given site by the number of individuals who had any positive response to the given vaccine insert. In order to obtain a conservative estimate of the response frequencies, given two consecutive positive 15 mer responses for a participant, we counted these as a single epitope by only tallying sites that were part of the overlap of the two peptides (typically 11 amino acids). Similarly, three and four consecutive peptides were considered as two distinct epitope responses, and five consecutive responses were counted as three distinct epitopes.

We also generated predicted population-based epitope maps, which were based on using HLA binding predictors. Specifically, we used the ADT algorithm, a structure-based epitope prediction method to predict epitopes for the HLA alleles of each vaccine cohort [30]. For these maps, we only considered predicted 9 mer epitopes. Similar to the experimentally measured maps, we identified all predicted epitopes for each vaccine insert and then tallied the number of reactive 9 mers that were predicted for each site along the protein, normalizing by the number of individuals in the cohort. For each clinical trial, predicted population-based maps were weighted by the HLA distribution of the trial participants. HLA binders were defined using a binding threshold on the IC50 value. We created maps using 3 thresholds: conservative (50 nM, strong binders), moderate (100 nM) and permissive (500 nM, weak binders).

### Statistical analysis

#### Comparing HLA distributions of different cohorts

HLA distributions of different cohorts were compared using Fisher's exact test for 2*n tables using R version 2.15.1.

### Testing for epitope hotspot existence

Epitope hotspots were defined as sets of contiguous sites that were targeted more frequently than under the null hypothesis of equal targeting frequencies for all sites. To assess the significance of such hotspots, we compared the targeting frequency of experimentally measured hotspots to those obtained from a null distribution in which the same number of epitopes were drawn at random from a uniform distribution for a given vaccine insert. Using 10,000 random realizations, p-values were computed for each hotspot as follows: hotspots were sorted in decreasing order of frequency and for each hotspot we computed the probability of obtaining a hotspot with similar or higher frequency under the null. We compared the first hotspot (highest peak) to the corresponding first hotspot in the random maps. Subsequent comparisons were done for all other peaks in descending frequency.

### Comparing population-based epitope maps

Two testing procedures were developed to test whether two epitope maps differ. These procedures compare the difference of two experimentally measured maps to those obtained from maps obtained by randomizing the cohort assignment of participants. If trial 1 had n1 participants and trial 2 had n2 participants, we randomly divided the n1+ n2 participants into two sets of size n1 and n2 and computed a population epitope map for these two randomly assigned sets. The first test statistic is based on the maximal difference in targeting frequency between two maps. Specifically, we computed the maximal difference between random maps for 10,000 pairs of maps and calculate p-values by comparing the frequency of obtaining maximal difference in frequency that was equal or larger than that obtained between the two observed maps. The second test statistic was based on the sum of absolute differences between two maps. Specifically, for each pair of maps we computed the absolute sum of differences in frequency. P-values are computed by comparing the set of differences between 10,000 pairs of random maps to the one obtained from the experimentally measured maps.

### Computing conservation scores

Conservation of sites across the HIV genome was computed using Shannon Entropy. Specifically, we used the LANL HIV entropy scores (computed using http://www.hiv.lanl.gov/content/sequence/NEWALIGN/align.html). Sites with low entropy are highly conserved, so negative scores were used for computing correlations with conservation, and for visualization.

### Vaccine targeting efficiency scores

The HLA targeting efficiency score is the Spearman correlation coefficient between binding scores and conservation scores for amino acids along a given protein. In Hertz et al. [23], these scores were computed for each HLA separately, and were based on predicted epitopes. Here, we computed vaccine targeting efficiency scores, which compute the correlation between experimentally measured population-based epitope maps and evolutionary conservation. A positive score indicates preferential targeting of conserved regions, and a negative score indicates preferential targeting of variable regions.

### Comparing conservation scores of hotspots vs. non-targeted sites

To compare the conservation scores of epitope hotpots vs. non-targeted sites, we defined epitope hotspots as sites that were targeted by more than 15% of the participants that had an epitope response to the given protein. Non-targeted sites were sites for which no epitope responses were detected.
